# Plasticity of Attentional Functions in Older Adults after Non-Action Video Game Training: A Randomized Controlled Trial

**DOI:** 10.1371/journal.pone.0092269

**Published:** 2014-03-19

**Authors:** Julia Mayas, Fabrice B. R. Parmentier, Pilar Andrés, Soledad Ballesteros

**Affiliations:** 1 Studies on Aging and Neurodegenerative Diseases Research Group, Department of Basic Psychology II, Universidad Nacional de Educación a Distancia, Madrid, Spain; 2 Neuropsychology and Cognition Group, Department of Psychology and Institute of Health Sciences (iUNICS), University of the Balearic Islands, Mallorca, Spain; 3 Instituto de Investigación Sanitaria de Palma (IdISPa), Mallorca, Spain; 4 School of Psychology, University of Western Australia, Perth, Australia; Catholic University of Sacred Heart of Rome, Italy

## Abstract

A major goal of recent research in aging has been to examine cognitive plasticity in older adults and its capacity to counteract cognitive decline. The aim of the present study was to investigate whether older adults could benefit from brain training with video games in a cross-modal oddball task designed to assess distraction and alertness. Twenty-seven healthy older adults participated in the study (15 in the experimental group, 12 in the control group. The experimental group received 20 1-hr video game training sessions using a commercially available brain-training package (*Lumosity*) involving problem solving, mental calculation, working memory and attention tasks. The control group did not practice this package and, instead, attended meetings with the other members of the study several times along the course of the study. Both groups were evaluated before and after the intervention using a cross-modal oddball task measuring alertness and distraction. The results showed a significant reduction of distraction and an increase of alertness in the experimental group and no variation in the control group. These results suggest neurocognitive plasticity in the old human brain as training enhanced cognitive performance on attentional functions.

**Trial Registration:**

ClinicalTrials.gov NCT02007616

## Introduction

Increasing evidence suggests that playing action video games enhances various aspects of cognition in young adults, including peripheral vision, visual attention, and spatial skills [1], [2], [3], [4], [5]. Most action video games are speeded, intense, violent, and may not be suitable for older adults, however. Interestingly, a recent study conducted with young adults showed that cognitive improvement is not limited to action games [6] and real time strategy-based video game training has been found to enhance executive control abilities in older adults [7]. It remains unknown, however, whether practicing non-action video games can be effective in enhancing other fundamental cognitive functions in older adults, such as for example attentional functions (which typically deteriorate in old age). In this study, we were interested in investigating whether the ability to filter out irrelevant information, that is, the ability to resist distraction by irrelevant stimuli, can improve in older adults after practicing non-violent video games.

Identifying factors that may shield individuals from cognitive deterioration in old age is of societal interest given the overall aging of the population observed in western societies and the continuous increase of life expectancy (as marked, for example, by the increasing number of centenarians). Although some cognitive functions, including world knowledge, verbal abilities [8], [9], [10] and implicit memory [11], [12], [13], [14], [15], [16], [17] are preserved in older adults, normal aging is associated with a significant decline in certain cognitive functions such as attention, episodic memory, working memory, processing speed, and executive functions [18], [19], [20], [21].

Attention is a fundamental building block of cognition and central to many mental functions. Evidence indicates that one important attentional function negatively affected by aging is the capacity to suppress irrelevant information to concentrate on the relevant task [22], [23]. Such findings fit with previous research showing that the prefrontal cortex (the dorsolateral prefrontal cortex in particular) is involved in attentional filtering [24], [25], [26], [27], [28], [29] and that these frontal regions usually undergo important changes in old age ([30], [28], [29]; but see [31]).

Recent years have seen a surge in attempts to identify ways to reduce and/or counteract the course of cognitive and brain decline. This trend is in good part based on the idea of cognitive and brain plasticity as a way of adaptation by compensatory neural activity [32], [33], [21]. The interest in developing computerized cognitive training programs to delay cognitive decline associated with aging has become more prominent [34], [35], [36]. One important issue in this field, however, is whether the effects of training are transferable to untrained tasks (so-called ‘transfer effect’). Despite the great appeal of video games as a way to improve perceptual and cognitive abilities, evidence of their efficacy is thus far mixed at best [37], [7], [3], [4], [35], [38], yet a recent meta-analytic study [39] suggests that video game training can be effective in healthy older adults but that its positive effects are moderated by several variables including the complexity of the video games, the age of the participants, the duration of the training, and the cognitive processes assessed. These authors concluded that: (a) old-old adults (70–80 years) benefit more from training than young-old adults (60–69 years); (b) training can be less effective is the number of sessions is very large; and (c) video game training enhances memory, attention, and speed of processing.

In this study we aimed at examining closer the effect of video game training on attention by measuring alertness and distraction in older adults. For this purpose we used a well specified and documented task, the cross-modal auditory-visual distraction oddball task [40], [41]. In this task, participants are presented with a sequence of visual digits that they must categorize as “odd” or “even” while ignoring irrelevant sounds preceding each target. In most trials the sound is the same (standard sound) while in a small proportion of the trials, interspersed at random, the standard sound is replaced by a specific alternative sound (deviant sound) or a new sound not presented earlier in the task (novel sound). Typically, participants in this task respond slower to visual stimuli when these follow a deviant or novel sound compared to the standard sound (deviance or novelty distraction). This type of distraction does not reflect the slower processing of the target stimuli but, instead, the time penalty associated with the involuntary orientation of attention to and from the deviant stimulus [42], [43] and, in certain conditions, the time required to resolve the conflict between the involuntary processing of the deviant sounds' content and the voluntary processing of the target stimulus [44], [45], [46].

Recent work indicates that deviance distraction relates to the involuntary use of the sound by the cognitive system to prepare for action [47], [48], [49] and that it occurs because deviant sounds violate the cognitive system's expectations rather than because such sounds are rare across the task [50], [51], [52]. From an electrophysiological perspective, deviant sounds elicit a triumvirate of rapid, specific, and automatic brain responses [53], [54], [55], [56], [57], [58]: mismatch negativity thought to reflect the mismatch between an incoming sound and past sounds (MMN) [59], [60], P3a thought to reflect the involuntary orientation toward the deviant [61], and the re-orientation negativity corresponding to the reorientation of attention toward the task at hand (RON) [62]. Of interest, these responses involve frontal areas [61], [63], [64], [65], which are known to suffer neuronal loss in old age [27], [29]. Phasic alertness is a useful cognitive function that prepares the organism for detecting and responding faster to an external signal. This improvement is related to the capacity of a warning signal to prepare the system for a rapid response [26]. In this study, we refer to alerting as the result of an involuntary increase in arousal produced by the auditory input, as the latter constitutes a warning signal announcing the imminent presentation of the target stimulus.

Using a version of the cross-modal oddball task including a silent block of trials, Andrés, Parmentier and Escera [40] (2006) compared deviance distraction (comparing performance in the deviant and standard conditions) and alertness (preparation for an upcoming stimulus; [66], measured by comparing performance in the silent and standard conditions) in young and older adults. Their results showed a two-fold increase in distraction in older compared to young adults while the two groups exhibited similar levels of alertness (a finding replicated by Parmentier and Andrés [41]; see also [67], for converging evidence in a unimodal auditory oddball task). The latter fits with the finding that alertness, while involving frontal structures, is supported by a more diffuse and distributed network including parietal and thalamic structures [68].

It remains unknown whether the ability to use sounds to prepare for action (phasic alertness) and to resist distraction by irrelevant oddball auditory distracters can improve in older adults through video game training. The present study investigated this issue using an intervention method. Intervention studies offer a controlled way to evaluate cognitive-enrichment changes because they use a controlled way to assign individuals to treatment conditions [33]. If video game training improves auditory attentional functions as it does visual attention [2] or executive functions [7], then older adults undergoing video game training in our study would exhibit reduced distraction and maintain their level of alertness or prevent its decline.

## Methods

### 1. Randomized controlled trial design

This randomized single blind controlled trial was conducted between January 2013 and July 2013 in Madrid (Spain). Experimenters implemented this trial. Participants were blind to the treatment and control persons that were assigned to each group. The researcher (JM) assigned participants to either of the two groups using the e-Prime software. Written informed consent to participate in the study was obtained from each participant. The protocol of this study and the informed consent were approved by the Ethics Committee of the Universidad Nacional de Educación a Distancia (UNED), Madrid (Spain). The protocols for this study and supporting CONSORT checklist are available as supporting information: See Checklist S1 and Protocol S1.

### 2. Participants

**Figure 1:**
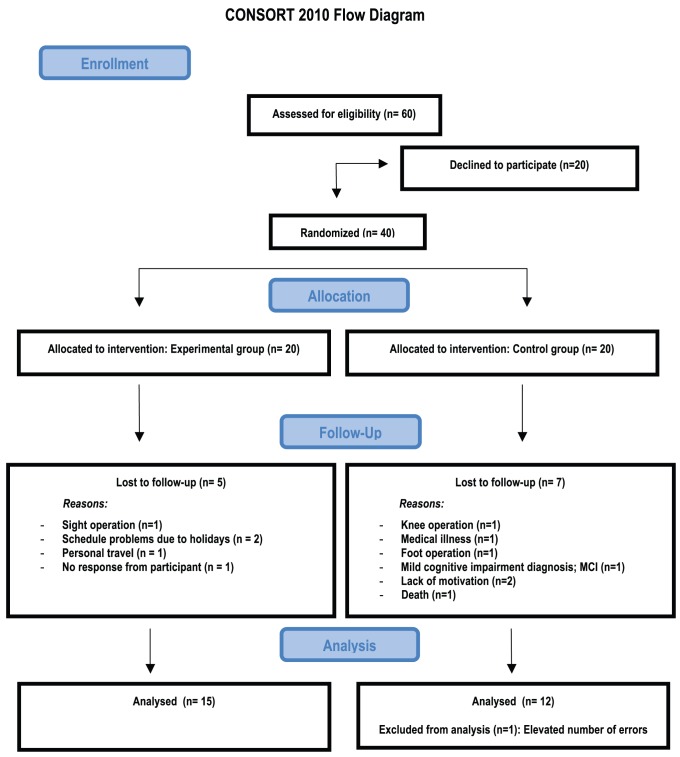
CONSORT flowchart.

Sixty healthy adult volunteers aged 57 to 77 years were recruited to participate in this study. Twenty participants declined to participate and the rest (40) were randomly assigned to the experimental and the control groups. The study was completed by 15 of the 20 participants of the experimental group and 12 of the 20 members of the control group (see Figure 1). All participants had normal hearing and normal or corrected-to-normal vision. In addition to the oddball task, all participants completed a battery of cognitive tests before the intervention: a) Mini-mental state examination (MMSE) [69], used to rule out possible cognitive impairment, b) Yesavage depression scale [70], used to identify possible participants suffering from depression, c) Vocabulary subscale from the WAIS [71]. Level of education was also registered. T-tests revealed no significant differences between the two groups (all *ps* >.05) for any of these measures prior to the training phase (see Table 1).

**Table 1:** Demographic data and mean test scores corresponding to trained older adults and controls.

*Group*	*Age*	*Female/Male*	*GDS*	*MMSE*	*Vocabulary*	*Education (years)*
Experimental (n = 15)	68.7 (5.2)	9/6	1.6 (1.2)	28.9 (1)	62.1 (9.9)	11.9 (4.8)
Control (n = 12)	68.5 (5.7)	4/8	2.5 (2.9)	28.8 (1)	60.1 (7.2)	13 (3.3)

*Note:* Standard deviation (SD); MMSE (Mini Mental State Examination /30); GDS (Geriatric Depression Scale /15).

All participants were informed of their right to terminate participation at any time, gave their written informed consent before participating in the study and were remunerated 75 € for their participation.

### 3. Design

All participants were tested individually prior to and following the intervention phase. The participants assigned to the training group underwent 20 laboratory-based sessions of video game training across a period of 10–12 weeks. Each session lasted approximately 60 min. These participants were trained in groups of three. We describe the video game training program below.

### 4. Training Program and Apparatus

The training sessions were performed in the laboratory. Participants taking part in the training sessions practiced 10 video games selected from the commercial package *Lumosity* (http://www.lumosity.com), each being practiced twice along 20 1-hr sessions (see Table 2). Video games were practiced in a random order. Participants practiced the games on a PC computer equipped with a 21-inch monitor. The games practiced were designed to train a variety of mental abilities, including speed of processing and mental rotation (*Speed match* and *Rotation matrix*), working memory (*Face memory*, *Memory match, Memory matrix, Moneycomb*), concentration (*Lost in migration* and *Space junk*), and mental calculation (*Raindrops* and *Chalkboard*). Points were awarded to participants based on their performance on the individual video games. The time taken by participants to complete a game was also recorded in four of the games (Memory match, Memory faces, Speed match and Lost in migration). Game performance was recorded for each participant in each session. No participant reported playing any other video game across the duration of our study. None of the participants reported any previous experience with video games. The control group did not receive video games training but participated in three group meetings during this time. In these meetings members of the research group discussed with the participants general topics related to aging and have coffee and drinks together.

**Table 2:** Mean Z-Scores (mean 0; standard deviation 1) obtained by the 15 participants in the 10 video games across the 20 training sessions.

TRAINING SESSION (MEAN SCORES)																				
GAME	1	2	3	4	5	6	7	8	9	10	11	12	13	14	15	16	17	18	19	20
**Raindrops**	−2,0	−1,6	−1,4	−0,7	−0,9	−0,6	−0,3	−0,7	−0,6	0,0	0,8	0,6	0,8	0,3	1,2	1,2	0,4	0,9	1,1	1,5
**M. Matrix**	−2,2	−1,7	−1,0	−0,8	−0,7	−0,4	−0,2	0,2	−0,4	−0,3	−0,7	1,2	0,5	0,6	0,6	0,4	1,1	1,7	1,5	0,6
**M. Match**	−1,7	−1,6	−1,2	−1,3	−1,0	−0,4	−0,2	−0,1	−0,2	−0,3	−0,1	0,9	−0,1	0,7	1,5	1,3	0,8	0,5	0,6	1,9
**Speed Match**	−2,1	−1,9	−1,5	−1,4	−0,5	−0,9	0,0	0,1	0,3	0,3	0,2	0,5	0,8	0,4	0,5	0,7	1,4	1,0	1,1	1,0
**Chalkboard**	−2,9	−1,9	−1,1	−0,7	−0,6	−0,3	−0,2	0,1	0,3	0,7	0,4	0,6	0,8	0,3	0,6	0,6	1,0	0,2	1,6	0,6
**Moneycomb**	−2,4	−2,1	−1,4	−0,2	−0,2	0,7	0,9	1,4	0,3	−0,1	−0,2	−0,6	1,1	−0,1	1,1	−0,2	0,7	0,1	1,0	0,2
**Rotation M.**	−2,4	−1,9	−1,1	−1,1	−0,4	0,0	−0,3	−0,3	−0,2	−0,1	0,0	1,0	1,3	0,7	0,9	0,5	0,8	0,4	0,6	1,6
**L. Migration**	−1,8	−1,6	−1,4	−1,4	−1,0	−0,7	−0,4	−0,5	0,1	0,4	0,1	0,5	0,6	0,8	0,8	0,9	0,9	1,2	1,1	1,5
**M. Faces**	−1,8	−1,5	−1,1	−1,1	−1,0	−0,9	−0,7	−0,6	0,0	0,0	0,1	0,3	0,8	0,7	0,9	0,8	1,1	1,1	1,5	1,4
**Space Junk**	−3,2	0,4	−1,1	1,1	−0,6	1,2	−0,6	0,4	−0,8	−0,9	0,8	−0,2	0,5	0,4	0,8	0,2	0,4	−0,3	0,9	0,6
**COMPLETION TIME (MEAN)**																				
**M.Match**	3,0	2,1	1,0	0,7	0,4	0,2	−0,3	−0,2	−0,4	−0,3	−0,4	−0,3	−0,3	−0,5	−0,7	−0,7	−0,8	−1,0	−0,8	−0,8
**Speed Match**	2,5	2,5	1,3	0,7	0,4	0,1	−0,1	−0,1	−0,4	−0,2	−0,1	−0,5	−0,6	−0,6	−0,7	−0,7	−0,9	−0,8	−0,9	−0,8
**L.Migration**	2,2	2,0	1,6	1,0	1,0	0,3	0,0	0,2	−0,4	−0,4	−0,2	−0,3	−0,7	−0,7	−0,8	−1,0	−0,9	−0,9	−1,1	−0,9
**Face**	3,8	1,3	0,5	0,3	0,4	0,0	−0,1	0,0	−0,1	−0,2	−0,3	−0,5	−0,5	−0,5	−0,7	−0,6	−0,7	−0,7	−0,7	−0,7

### 5. Stimuli and Procedure: Oddball task

Stimulus presentation and data collection were performed using a bespoke program written in E-Prime 2.0 (Psychology Software Tools Inc, Pittsburg, PA, USA). Participants were tested individually in a quiet room.

Participants performed three blocks of 384 trials each (24 practice trials and 360 test trials, as described below). In each trial, participants categorized a visual digit (1–8) as odd or even by pressing response keys (V & B, counterbalanced across participants) with two fingers from their dominant hand. Each trial began with the presentation of a white fixation cross at the centre of the black screen (the cross remained visible throughout the trial except when the digit was presented) as well as a 200 ms sound. The visual digit (in white color) appeared at the center of the screen 100 ms after the sound's offset, and remaining on the screen for 200 ms. The digit's size corresponded to a viewing angle of approximately 2.6 °. A response window of 1200 ms was available from the digit's onset.

Three sound conditions were compared, defined by the content of the sound file played in each trial. In the silent condition, participants performed a whole block of trials in which each trial used a silent sound file. The remaining two blocks contained two types of sounds. The standard sound, used in 576 trials (80%), was a 600 Hz sinewave tone of 200 ms in duration (10 ms of rise/fall times), digitally recorded and low-pass filtered at 10,000 Hz. In the remaining 144 (20%) trials, novel sounds were used. These sounds were taken from a list of 72 novel environmental sounds (e.g., hammer, drill, door, rain, etc.) obtained from Andrés et al. [40], with each sound used twice across the task. All sounds were normalized and presented binaurally through headphones at approximately 75 dB SPL. Half the participants completed the silent block followed by two sound blocks while the other half completed the two sound blocks first, followed by the silent block. Across the task, each digit was used with equal probability in each of the sound conditions.

Participants were instructed to focus on the digit categorization task and ignore any sound presented in order to respond to the digit as accurately and rapidly as possible.

### 6. Statistical Analyses

Two independent 2 (group: experimental *vs* control, between-participants) x 2 (time of assessment: pre *vs* post, within-participant) x 3 (sound: silence, standard, novel, within-participant) mixed-model ANOVA was carried out on the mean of accuracy and on the mean of RTs for correct responses.

## Results

In order to determine whether training improved performance on the video games, we first analyzed the effect of training for each of the 10 video games. We then analyzed performance in the oddball task to examine whether the video game training impacted on alertness and distraction.

### 1. Video game performance

Table 2 displays the mean performance on Z-Scores of the group who trained on the 10 video games across the 20 training sessions. The overall performance on the games improved as a function of session both in terms of accuracy and completion time (in those video games that registered that variable).

Comparisons between the first and last sessions were carried out on the performance Z-score. The results showed that training increased performance, both in terms of accuracy as well as in completion times (see Table 3).

**Table 3:** Performance (mean scores and completion time) of the experimental group on the first and last training session in each of the 10 non-action video games.

*GAME*	*t (14)*	*p-value*
Raindrops	5.3	<.001
M. Matrix	5.5	<.001
M.Match	2.6//4.9	= .017//<.001
Speed match	6.3//3.7	<.001// = .002
Chalkboard	3.6	= .002
Moneycomb	2.4	= .026
Rotation M.	4.5	<.001
L. Migration	4.3//10.5	= .001//<.001
M.Faces	6.3//5.3	<.001//<.00<
Space Junk	3.2	= .006

*Note:* Reaction time (RT); Performance on the 10 games improved significantly with training. Reaction times improved significantly in the 4 games that registered velocity.

### 2. Effect of video game training on performance in the oddball task

Accuracy levels in the digit categorization task in both groups were similar [*F*(1,25) = 1,38, *MSE* = 0.05, *p* = .25, 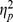 = 0.05]. The mean proportion of correct responses in the experimental group was .90 (*SD* = 0.05) and .92 (*SD* = 0.04) for pre and post-test, respectively, and .90 (*SD* = 0.05) and .90 (*SD* = 0.02) in the control group for pre and post-test, respectively. A significant effect of condition was observed, [*F*(2,50) = 5.52, *MSE* = 0.001, *p* = .007, 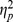 = 0.18], reflecting a lower proportion of correct responses in the novel condition compared to the silent and standard conditions, [*F*(1,25) = 7.087, *MSE* = 0.0013, *p*<.05, d = 0.15, and *F*(1,25) = 5.99, *MSE* = 0.0005, *p*<.05, d = .09] respectively, while the standard and silent conditions did not differ from each other, [*F*(1,25) = 2.21, *MSE* = 0.0007, *p* = .150, d = 0.06]. The main effect of the time of assessment was not statistically significant [*F*(1,25) = 2.25, *MSE* = 0.0025, *p* = .150, 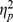 = 0.08]. None of the two-way interactions were significant: Group x time of assessment [*F*(1,25)<1, *MSE* = 0.0491, *p* = .438, 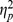 = 0.02], group x condition [*F*(2,50)<1, *MSE* = 0.0008, *p* = .907, 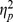 = 0.04], and condition x time of assessment [*F*(2,50)<1, *MSE* = 0.0007, *p* = .542, 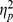 = 0.02]. Finally, the three-way interaction group x time of assessment x condition was marginally significant, [*F*(2,50) = 2.71, *MSE* = 0.0007, *p* = .077, 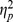 = 0.09].

Response times for correct responses were analyzed after removing outliers (RTs faster than 200 and slower than 1100 ms, representing 3.6% and 3.04% of the trials for pre- and post-training respectively). Standard trials immediately following novel trials were excluded from the analysis, as these have been shown to yield residual distraction [72].

**Figure 2:**
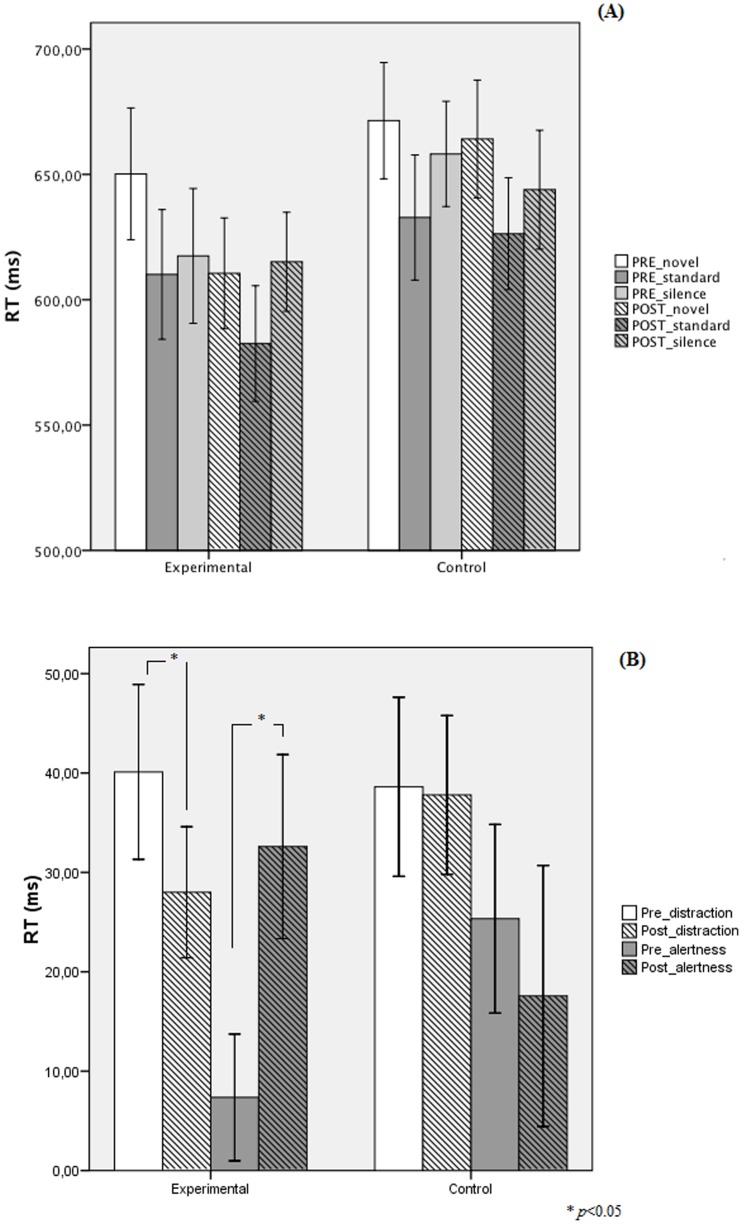
Performance of experimental (trained) and control group participants. Panel A: Mean response times for each group at pre and post-training in the three experimental conditions (silence, standard and novel). Panel B: Mean alertness and distraction effects for trained and controls participants.

This analysis revealed that the main effect of group was not statistically significant [*F*(1,25) = 1.2, *MSE* = 40075.19, *p* = .27, 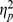 = 0.04]. The mean RTs were 614 and 649 ms for the experimental and control groups, respectively. The main effect of condition was significant [*F*(2,50) = 26.63, *MSE* = 657.99, *p* = .000, 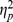 = 0.51]. Pairwise comparisons showed that there were statistical significant differences between the three conditions (*p*<.05). The mean RT corresponding to the standard condition (613 ms) was the fastest, followed by the mean RT corresponding to the silence condition (634 ms), being the slowest the RT corresponding to the novel condition (649 ms). The main effect of time of assessment was not statistically significant, [*F*(1,25) = 2.56, *MSE* = 4126.29, *p* = .12, 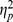 = 0.09]. The two-way interaction group x time of assessment [*F*(1,25) = 0.46, *MSE* = 4126.29, *p* = .50, 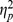 = 0.01], group x condition [*F*(2,50) = 0.09, *MSE* = 403.54, *p* = 0.15, 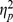 = 0.00] and time of assessment x condition [*F*(2,50) = 1.92, *MSE* = 403.54, *p* = 0.15, 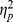 = 0.13] were not significant. Most critically, the triple interaction, depicted in Figure 2 (Panel A) was statistically significant [*F*(2,50) = 4.37, *MSE* = 403.54, *p* = 0.018, 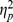 = 0.14]. This interaction was analyzed using contrasts to assess the extent to which distraction (novel versus standard) and alertness (silence versus standard) [40], [41] varied in each group as a function of the time of assessment (see Figure 2, Panel B).

#### Distraction

A comparison pre- and post-training conditions showed a significant reduction of distraction in the experimental group [*F*(1,25) = 4.00, *MSE* = 474.28, *p* = .05, d = 0.43] but not in the control group [*F*(1,25) = 0.001, *MSE* = 274.28, *p* = .970, d = 0.03. In other words, the ability to ignore irrelevant sounds improved significantly in the experimental group after video game training (12 ms approximately) but not in the control group.

#### Alertness

Pre- and post-training comparisons showed a 26 ms increase of alertness in the experimental group [*F*(1,25) = 4.45, *MSE* = 1071.07, *p* = .04, d = 0.9] and no significant difference in the control group [*F*(1,25) = 0.387, *MSE* = 1071.07, *p* = .54, d = 0.22].

Finally, although our sample included only 15 participants practicing the video games, we explored the correlations between the improvement on each of the 10 games (computing by the difference between post and pre values) on the one hand, and distraction and alertness on the other hand. We found a marginal negative correlation between distraction and response times on the “memory speed” game (*r* = −0.49; *p* = .058), a significant negative correlation between distraction and response time in the “speed match” game (*r* = −0.56; *p* = .02). Alertness showed a positive correlation (*r* = 0.6; *p*<.01) with response times on the “Lost in migration” game. All other correlations were non-significant.

## Discussion

In the present study, we used a cross-modal oddball paradigm to investigate whether cognitive training with non-action video games would improve older adults' performance in two basic attentional functions: (1) filtering out task-irrelevant information to maintaining the focus of attention on task-relevant information; and (2) alertness (to capitalize on the presentation of a warning cue to prepare for action). The experimental (trained) and control groups performed the oddball task with similar levels of accuracy at the first (pre-test) and the second assessments (post-test). However, response times showed benefits of the video game training, as reflected by the increase in alertness and the reduction of deviance distraction.

Unexpected deviant sounds distract participants away from a focal visual task [43], [42]. Such distraction is stronger in older compared to younger adults [40], [41], [67], [73], [74], [75], [76]. The results of the present intervention study show for the first time that distraction can be reduced in older adults through brain training using video games, therefore highlighting such practice as a potential protective factor against the effect of cognitive aging. The second key finding of our study was the positive effect of brain training on alertness, an attentional function some argued is affected by age [77], [78] while others argued it is not [40].

Although age is accompanied by cortical deterioration (especially of the prefrontal region [79], [80], [28], [29], there is evidence that neuroplasticity (the physiological capacity of the brain to form and strengthen neuronal connections) is not limited to early years but extends to old age [81], [82], [83]. Neuroplasticity not only brings morphological changes but may also increase cognitive reserve [84], [85], [83], [86]. As part of growing interest in the mechanisms involved in cognitive and neural plasticity across the lifespan [32], [87], [21], [83], one important issue relates to the potential modifiability of cognitive function during the old age and, ultimately, to the possibility of helping older individuals to maintain longer quality of life and independence. In this context, numerous contributions have provided evidence for the utility of different training programs to improve memory skills [88], [89], [90], [91], executive, attention and inhibition functions [82], [92], [6] and processing speed [92], [35]. Our results add to this body of literature by suggesting that attentional filtering and alertness too can be enhanced through cognitive training. It also supports the idea that cognitive training based on *Lumosity* benefits some aspects of cognitive performance in old age [93], [94].

One aspect of our study worth emphasizing is that the video games we used were distinct from the task we used to measure filtering and alertness. That is, the benefits of the training manipulation was not only limited to performance on these games but also transferred to the oddball task. An interesting question is why training with these video games can transfer to these attentional functions. One possibility is that practicing the games improved sustained attention and concentration in general, rather than any specific information processing skill per se. That is, practicing the games might have helped participants to maintain attention and optimize its efficiency (e.g., resisting to deviations of attention in response to task-irrelevant information).

In the present study, we observed this effect using non-violent and non-action video games that may be more appealing to older adults. Practicing video games of this type may offer some protective factor against the effects of aging and may potentially be recommended to older individuals, alongside other interventions found to improve mental functions. These include, for example, a long-term physically active lifestyle (improving executive control and speed of processing, [95]), aerobic exercise (improving cognition by increasing the volume of grey and white matter in frontal and temporal sites, [96], [97]), or social networking and innovative solutions to connect people in a multimodal way with family members, friends and caretakers [98], [99].

In summary, research on the benefits of video games has experienced a recent surge among investigators interested in training the cognitive abilities of older adults to prevent cognitive decline, making it an important topic for psychologists, gerontologists and neuroscientists. The results of the present study suggest that training older adults with non-action video games reduces distractibility by improving attention filtering (a function declining with age and largely depending on frontal regions) but also improves alertness. Whether similar changes in alertness and distraction after cognitive training might be observed in younger participants, who are typically regarded to be at the peak of cognitive functioning, is an interesting and open question that would deserve further investigation.

Our study presents some limitations. One is the small sample size. Although we recruited sixty participants at the beginning of the study, only 40 volunteers agreed to participate in this intensive longitudinal study. In the final analyses only twenty-seven participants completed the study and were included in the analysis. A second limitation of the present study is that does not examine whether the impact of video game training may generalize to everyday life tasks. Further research should address whether this improvement would transfer to real world tasks. It is also worth pointing out that the games forming part of the Lumosity suite could be described as tasks similar to some found in intelligence test batteries. Hence another possible avenue for research will be to determine whether the benefits of such games may generalize to video games more similar to those found in the gaming market. Finally, another criterion regarding the importance of the cognitive training results is whether there is evidence of endurance of the training effects. Future research should evaluate the maintenance of training effects.

## Supporting Information

Checklist S1 **CONSORT Checklist.**
(PDF)

Protocol S1 **Trial Protocol.**
(PDF)
