# Supplementary material for: Plasticity of Attentional Functions in Older Adults after Non-Action Video Game Training: A Randomized Controlled Trial
Source: PLoS One. 2014 Mar 19;9(3):e92269. doi: 10.1371/journal.pone.0092269 (PMC3960226; doi:10.1371/journal.pone.0092269)
Supplement: Protocol S1 — Trial Protocol. (PDF) [file pone.0092269.s002.pdf]

Plasticity of attentional functions in older adults

after non-action video game training: A randomized controlled trial

Trial Protocol

**Title**	Plasticity of attentional functions in older adults<br>after non-action video game training: A randomized controlled trial
**Summary**	The aim of this trial is to investigate whether older adults could benefit from brain training with non-nvideo games (20 sessions of 1-hour during three months) in a cross-modal oddball task designed to assess distraction and alertness.
**Study Design**	Ramdomized controlled trial with trained and control groups
**Description**	A major goal of recent research in aging has been to examine cognitive plasticity in older adults and its capacity to counteract cognitive decline. The aim of the present study was to investigate whether older adults could benefit from brain training with non-action video games in a cross-modal oddball task designed to assess distraction and alertness. Two groups of healthy older adult volunteers participated in the study. The experimental group received 20 1-hr video game training sessions using a commercially available brain-training package (*Lumosity)* involving problem solving, mental calculation, working memory and attention tasks. The control group did not practice this package and, instead, attended meetings with the other members of the study several times along the course of the study. Both groups were evaluated before and after the intervention using a cross-modal oddball task measuring alertness and distraction. The results showed a significant reduction of distraction and an increase of alertness in the experimental group and no variation in the control group. These results suggest neurocognitive plasticity in the old human brain as training enhanced cognitive performance on attentional functions.
**Outcome measure(s)**	Neuropsychological testing<br>Cross-modal (visual – auditory) Oddball attentional task
**Sample Size**	See Consort flowchart
**Setting**	Universidad Nacional de Educación a Distancia, Madrid (Spain)
**Project coordinator**	Profesor Soledad Ballesteros<br>Principal Investigator of the *Studies on Aging and Neurodegenerative Diseases Research Group*, Department of Basic Psychology II, Universidad Nacional de Educación a Distancia, Madrid (Spain)

**Overall Study Official(s)**	Universidad Nacional de Educación a Distancia (UNED) and University of the Balearic Islands<br>Spain
**Human subjects Review/Oversight**	This research was approved by the Ethics Committee of UNED, institution in which the study was conducted.
**Collaborators**	Julia Mayas*, Fabrice B. R. Parmentier**, ***, Pilar Andrés**, and Soledad Ballesteros*,<br>**Studies on Aging and Neurodegenerative Diseases Research Group*, Department of Basic Psychology II, Universidad Nacional de Educación a Distancia, Madrid (Spain)<br>** Department of Psychology and Institute of Health Sciences (iUNICS), University of the Balearic Islands, Mallorca (Spain)<br>*** Instituto de Investigación Sanitaria de Palma (IdISPa)*<br>*** School of Psychology, University of Western Australia, Perth (Australia)
**Funding Source**	This work was supported by grants from the Spanish Government to S. Ballesteros (PSI2010-21609-C02-01) and P. Andrés (PSI2010-21609-C02-02), Madrid Community Biomedical Research grant to S. Ballesteros (S2010/BMD-2349), as well as a research grant from the Spanish Ministry of Science and Innovation (PSI-2009-08427) and Plan E and a Ramón y Cajal Fellowship (RYC-2007-00701) awarded to Fabrice Parmentier.
